# Supplementary material for: The Type of Fat in the Diet Influences Regulatory Aminopeptidases of the Renin-Angiotensin System and Stress in the Hypothalamic-Pituitary-Adrenal Axis in Adult Wistar Rats
Source: Nutrients. 2021 Nov 4;13(11):3939. doi: 10.3390/nu13113939 (PMC8625891; doi:10.3390/nu13113939)
Supplement: Supplementary file 1 [file nutrients-13-03939-s001.zip › Supplementary matterial.pdf]

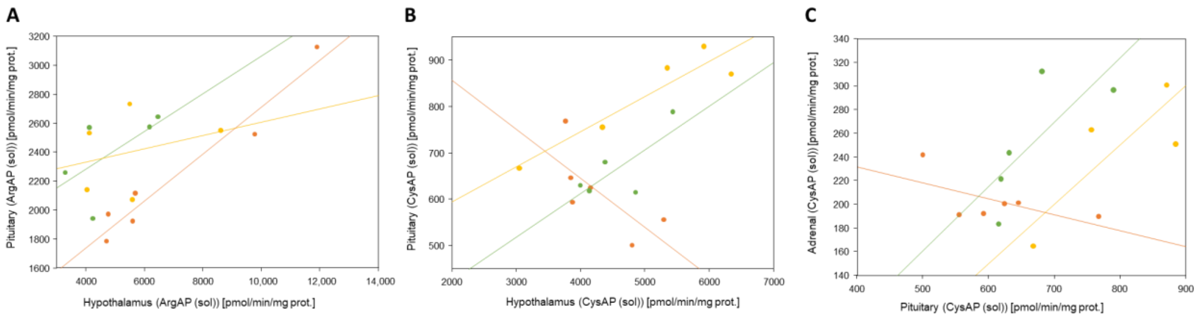

Intra-group correlation:	S – S	VOO – VOO	Bch – Bch
P-value:	0.001 **	0.273	0.625
R:	0.973	0.612	0.299

Intra-group correlation:	S – S	VOO – VOO	Bch – Bch
P-value:	0.107	0.141	0.020 *
R:	0. 720	0.753	0.934

Intra-group correlation:	S – S	VOO – VOO	Bch – Bch
P-value:	0.186	0.148	0.054
R:	0.623	0.746	0.872

Supplementary Figure S1. Dietary intra-group correlations between significant (A) arginyl-aminopeptidase (ArgAP) and (B-C) cystinyl aminopeptidase (CysAP) activities analyzed in soluble (sol) fractions of (A-B) hypothalamus – pituitary [pmol/min/mg prot.], and CysAP (sol) activity analyzed in (C) pituitary - adrenal glands [pmol/min/mg prot.]. P-value less than 0.05 was considered significant, being *p <0.05 and **p <0.01. R: linear correlation coefficient. The table below the figure represents the correlations between different tissues with the same dietary groups (intra-group correlation). S: standard diet; VOO: virgin olive oil diet; Bch: butter plus cholesterol diet.

Supplementary Table S1. Angiotensinase activities at hypothalamus-pituitary-adrenal axis.

Sample	Angiotensinase	Diet	Mean ± SEM (sol) [pmol/min/mg prot.]	P-value	Mean ± SEM (mb) [pmol/min/mg prot.]	P-value
Hypothalamus	AspAP	S	711.81 ± 120.84	0.468	538.85 ± 94.41	0.353
		VOO	545.71 ± 47.61		413.25 ± 45.01	
		Bch	632.02 ± 78.84		472.07 ± 40.50	
	GluAP	S	519.41 ± 28.04	0.610	627.59 ± 84.62	0.504
		VOO	537.63 ± 58.73		529.60 ± 45.81	
		Bch	593.73 ± 64.22		617.70 ± 28.28	
	AlaAP	S	9393.84 ± 819.40	0.212	4891.07 ± 352.48	0.052
		VOO	7667.87 ± 607.39		4090.26 ± 149.91	
		Bch	9521.10 ± 811.38		3971.51 ± 189.33	
	ArgAP	S	7085.17 ± 1233.01	0.285	4274.49 ± 277.94	0.661
		VOO	4869.74 ± 619.63		4244.15 ± 380.20	
		Bch	5566.53 ± 827.14		3927.90 ± 176.54	
	CysAP	S	4294.59 ± 253.68	0.449	690.28 ± 88.01	0.561
		VOO	4570.40 ± 262.12		595.02 ± 40.48	
		Bch	4997.03 ± 591.41		663.38 ± 23.24	
Pituitary gland	AspAP	S	93.43 ± 9.77	0.762	443.72 ± 41.89	0.358
		VOO	104.09 ± 13.57		404.36 ± 29.07	
		Bch	101.29 ± 9.07		497.64 ± 54.14	
	GluAP	S	386.90 ± 15.98	0.376	3494.81 ± 120.41	0.105
		VOO	430.91 ± 31.97		3371.35 ± 184.42	
		Bch	420.80 ± 20.64		4247.77 ± 470.35	
	AlaAP	S	2027.08 ± 180.69	0.001	4120.29 ± 286.42	0.024
		VOO	2361.64 ± 112.38	#0.030	4156.58 ± 227.06	#0.048
		Bch	2955.28 ± 75.31$^{\#*}$	*0.001	5553.27 ± 522.90$^{\#*}$	*0.034
	ArgAP	S	2237.90 ± 204.96	0.732	2876.15 ± 299.72	0.029
		VOO	2393.09 ± 131.98		3023.28 ± 47.22	#0.035
		Bch	2400.90 ± 127.75		3810.46 ± 213.85	*0.012
	CysAP	S	614.03 ± 37.24	0.007	1205.01 ± 119.84	0.505
		VOO	666.70 ± 32.79	#0.046	1297.38 ± 89.44	
		Bch	822.35 ± 48.02$^{\#*}$	*0.006	1389.92 ± 112.36	
Adrenal gland	AspAP	S	18.19 ± 0.45	0.041	89.24 ± 2.80	0.172
		VOO	28.33 ± 3.79*	*0.027	105.04 ± 12.12	
		Bch	27.23 ± 3.81*	*0.034	125.12 ± 20.14	
	GluAP	S	95.87 ± 3.15	0.336	878.17 ± 44.97	0.779
		VOO	114.36 ± 11.47		871.44 ± 75.73	
		Bch	115.05 ± 14.77		938.14 ± 93.88	
	AlaAP	S	551.32 ± 16.64	0.589	1378.89 ± 114.46	0.541
		VOO	619.92 ± 52.53		1485.66 ± 187.29	
		Bch	583.29 ± 66.18		1654.77 ± 224.82	
	ArgAP	S	379.58 ± 20.59	0.154	872.57 ± 47.09	0.114
		VOO	536.17 ± 70.70		951.16 ± 102.99	
		Bch	501.09 ± 75.52		1229.02 ± 181.19	
	CysAP	S	203.03 ± 8.01	0.123	358.41 ± 10.92	0.151
		VOO	251.49 ± 23.83		430.14 ± 41.09	
		Bch	261.25 ± 27.70		476.42 ± 62.72	

Note: The values represent means and standard error of the mean (SEM) of angiotensinases activities in soluble (sol) and membrane-bound (mb) fractions of hypothalamus, pituitary and adrenal glands atrium [pmol/min/mg prot.]. AlaAP: alanyl aminopeptidase; ArgAP: arginyl aminopeptidase; AspAP: aspartyl aminopeptidase; CysAP: cystinyl aminopeptidase; GluAP: glutamyl aminopeptidase. P-value less than 0.05 was considered significant. * $p < 0.05$, indicates significant differences between virgin olive oil diet (VOO) or butter plus cholesterol diet (Bch) vs. standard diet (S). # $p < 0.05$, indicates significant differences between VOO and Bch.

Supplementary Table S2. Significative inter- and intra-gland correlations.

Inter-gland correlations	Angiotensinase activity (fraction)	Correlation coefficient (R)	P-value
Hypothalamus – Pituitary	ArgAP (sol) vs. ArgAP (sol)	0.616	0.011*
	CysAP (sol) vs. CysAP (sol)	0.522	0.038*
Pituitary – Adrenal	CysAP (sol) vs. CysAP (sol)	0.616	0.011*
**Intra-gland correlations**	**Angiotensinase activity (fraction)**	**Correlation coefficient (R)**	**P-value**
Hypothalamus	AlaAP (sol) vs. ArgAP (sol)	0.679	$3.86*10^{-3}$ **
	AlaAP (sol) vs. AspAP (sol)	0.636	$8.09*10^{-3}$ **
	AlaAP (sol) vs. CysAP (sol)	0.596	0.015*
	ArgAP (sol) vs. AspAP (sol)	0.951	$1.56*10^{-8}$ ***
	ArgAP (sol) vs. GluAP (sol)	0.714	0.014*
	AspAP (sol) vs. GluAP (sol)	0.968	$1.05*10^{-6}$ ***
	CysAP (sol) vs. GluAP (sol)	0.640	0.034*
	AlaAP (mb) vs. AspAP (mb)	0.799	$2.06*10^{-4}$ ***
	AlaAP (mb) vs. CysAP (mb)	0.788	$2.87*10^{-4}$ ***
	AlaAP (mb) vs. GluAP (mb)	0.748	$8.62*10^{-4}$ ***
	AspAP (mb) vs. CysAP (mb)	0.983	$9.215*10^{-12}$ ***
	AspAP (mb) vs. GluAP (mb)	0.943	$4.69*10^{-8}$ ***
	CysAP (mb) vs. GluAP (mb)	0.969	$6.77*10^{-10}$ ***
Pituitary	AlaAP (sol) vs. CysAP (sol)	0.804	$1.72*10^{-4}$ ***
	ArgAP (sol) vs. AspAP (sol)	0.720	0.013*
	ArgAP (sol) vs. GluAP (sol)	0.647	$6.73*10^{-3}$ **
	AspAP (sol) vs. GluAP (sol)	0.811	$2.47*10^{-3}$ **
	AlaAP (mb) vs. ArgAP (mb)	0.654	$5.96*10^{-3}$ **
	AlaAP (mb) vs. AspAP (mb)	0.780	$3.61*10^{-4}$ ***
	AlaAP (mb) vs. CysAP (mb)	0.757	$6.94*10^{-4}$ ***
	AlaAP (mb) vs. GluAP (mb)	0.715	$1.83*10^{-3}$ **
	AspAP (mb) vs. CysAP (mb)	0.760	$6.27*10^{-4}$ ***
	AspAP (mb) vs. GluAP (mb)	0.804	$1.72*10^{-4}$ ***
	CysAP (mb) vs. GluAP (mb)	0.501	0.048*
Adrenal	AlaAP (sol) vs. ArgAP (sol)	0.847	$3.48*10^{-5}$ ***
	AlaAP (sol) vs. AspAP (sol)	0.750	$1.28*10^{-3}$ **
	AlaAP (sol) vs. CysAP (sol)	0.847	$3.44*10^{-5}$ ***
	AlaAP (sol) vs. GluAP (sol)	0.818	$1.07*10^{-4}$ ***
	ArgAP (sol) vs. AspAP (sol)	0.957	$2.47*10^{-8}$ ***
	ArgAP (sol) vs. CysAP (sol)	0.922	$3.85*10^{-7}$ ***
	ArgAP (sol) vs. GluAP (sol)	0.934	$1.22*10^{-7}$ ***
	AspAP (sol) vs. CysAP (sol)	0.926	$7.66*10^{-7}$ ***
	AspAP (sol) vs. GluAP (sol)	0.918	$1.35*10^{-6}$ ***
	CysAP (sol) vs. GluAP (sol)	0.925	$2.91*10^{-7}$ ***
	AlaAP (mb) vs. ArgAP (mb)	0.833	$6.06*10^{-5}$ ***
	AlaAP (mb) vs. AspAP (mb)	0.850	$3.00*10^{-5}$ ***
	AlaAP (mb) vs. CysAP (mb)	0.889	$4.07*10^{-6}$ ***
	AlaAP (mb) vs. GluAP (mb)	0.911	$9.08*10^{-7}$ ***
	ArgAP (mb) vs. AspAP (mb)	0.911	$9.18*10^{-7}$ ***
	ArgAP (mb) vs. CysAP (mb)	0.880	$6.83*10^{-6}$ ***
	ArgAP (mb) vs. GluAP (mb)	0.714	$1.87*10^{-3}$ **
	AspAP (mb) vs. CysAP (mb)	0.980	$3.52*10^{-11}$ ***
	AspAP (mb) vs. GluAP (mb)	0.762	$5.96*10^{-4}$ ***

CysAP (mb) vs. GluAP (mb)	0.805	$1.69*10^{-4}$ ***

Note: Correlation of the angiotensinase activities of the tissues (intra-gland correlations) and between the tissues (inter-gland correlations) that form the hypothalamic-pituitary-adrenal axis. AlaAP: alanyl-aminopeptidase; ArgAP: arginyl-aminopeptidase; AspAP: aspartyl-aminopeptidase; CysAP: cystinyl-aminopeptidase; GluAP: glutamyl aminopeptidase. Asterisks represent p-value * <0.05, ** <0.01, *** <0.001.

Supplementary Table S3. Dipeptidyl peptidase IV, Pyroglutamyl-aminopeptidase and Tyrosyl-aminopeptidase activities at hypothalamus-pituitary-adrenal axis.

Sample	Peptidase	Diet	Mean ± SEM (sol) [pmol/min/mg prot.]	P-value	Mean ± SEM (mb) [pmol/min/mg prot.]	P-value
Hypothalamus	DPP-IV	S	1363.16 ± 150.74	0.563	1315.03 ± 185.79	0.491
		VOO	1164.33 ± 133.71		1098.44 ± 138.18	
		Bch	1206.69 ± 120.74		1072.77 ± 122.81	
	pGluAP	S	767.46 ± 137.17	0.492	536.22 ± 89.01	0.179
		VOO	586.11 ± 55.63		422.10 ± 42.22	
		Bch	656.84 ± 94.00		453.95 ± 36.24	
	TyrAP	S	6257.55 ± 414.42	0.228	1515.91 ± 92.76	0.248
		VOO	6821.38 ±558.94		1741.36 ± 116.73	
		Bch	7850.25 ± 895.15		1667.63 ± 64.29	
Pituitary gland	DPP-IV	S	532.96 ± 28.05	0.046	1796.14 ± 172.24	0.174
		VOO	603.98 ± 41.97		1793.03 ± 124.54	
		Bch	651.73 ± 17.93*	*0.040	2255.74 ± 241.66	
	pGluAP	S	301.52 ± 15.85	0.337	1140.96 ± 127.87	0.685
		VOO	344.59 ± 28.51		1223.51 ± 93.63	
		Bch	338.76 ± 22.19		1279.10 ± 106.10	
	TyrAP	S	2504.09 ± 195.97	0.043	2046.39 ± 172.69	0.111
		VOO	2377.77 ± 203.59	#0.050	2067.54 ± 71.14	
		Bch	3072.27 ± 113.57#		2543.80 ± 227.75	
Adrenal gland	DPP-IV	S	517.03 ± 18.41	0.102	4314.22 ± 251.58	0.180
		VOO	625.91 ± 79.10		6300.32 ± 1381.92	
		Bch	723.86 ± 84.75		7916.71 ± 1981.50	
	pGluAP	S	21.54 ± 0.87	0.212	66.45 ± 2.76	0.116
		VOO	27.83 ± 3.28		93.02 ± 10.99	
		Bch	27.18 ± 3.62		115.81 ± 24.07	
	TyrAP	S	443.46 ± 12.66	0.120	507.53 ± 26.45	0.559
		VOO	513.04 ± 46.01		572.76 ± 70.74	
		Bch	617.06 ± 91.35		608.86 ± 98.00	

Note: The values represent means and standard error of the mean (SEM) of Dipeptidyl peptidase IV (DPP-IV), Pyroglutamyl-aminopeptidase (pGluAP) and Tyrosyl-aminopeptidase (TyrAP) activities in soluble (sol) and membrane-bound (mb) fractions of hypothalamus, pituitary and adrenal glands atrium [pmol/min/mg prot.]. P-value less than 0.05 was considered significant. * $p < 0.05$, indicates significant differences between virgin olive oil diet (VOO) or butter plus cholesterol diet (Bch) vs. standard diet (S). # $p < 0.05$, indicates significant differences between VOO and Bch.

Supplementary Table S4. Neuromarker Prolyl-iminopeptidase activity at hypothalamus and pituitary.

Sample	Peptidase	Diet	Mean ± SEM (sol) [pmol/min/mg prot.]	P-value	Mean ± SEM (mb) [pmol/min/mg prot.]	P-value
Hypothalamus	PIP	S	1371.38 ± 108.73	0.484	721.65 ± 86.28	0.416
		VOO	1234.07 ± 72.39		611.21 ± 39.50	
		Bch	1427.61 ± 139.07		723.89 ± 45.58	
Pituitary	PIP	S	430.85 ± 15.78	0.034	1137.57 ± 121.96	0.754
		VOO	476.32 ± 28.56		1202.27 ± 94.85	
		Bch	520.04 ± 19.99*	*0.027	1256.44 ± 112.11	

Note: The values represent means and standard error of the mean (SEM) of the neuromarker activity Prolyl-iminopeptidase (PIP) in soluble (sol) and membrane-bound (mb) fractions of hypothalamus and pituitary [pmol/min/mg prot.]. P-value less than 0.05 was considered significant. * $p < 0.05$, indicates significant differences between virgin olive oil diet (VOO) or butter plus cholesterol diet (Bch) vs. standard diet (S).

Supplementary Table S5. Significative inter-correlations between stress-related activities with renin-angiotensin system activities at hypothalamus-pituitary-adrenal axis.

Sample	Inter-correlation of activities (fraction)	Correlation coefficient (R)	P-value
Hypothalamus	DPP-IV (sol) vs. AlaAP (sol)	0.674	$4.17*10^{-3}$ **
	DPP-IV (sol) vs. ArgAP (sol)	0.847	$3.45*10^{-5}$ ***
	DPP-IV (sol) vs. AspAP (sol)	0.885	$5.38*10^{-6}$ ***
	DPP-IV (sol) vs. GluAP (sol)	0.857	$7.50*10^{-4}$ ***
	DPP-IV (sol) vs. pGluAP (sol)	0.892	$7.86*10^{-6}$ ***
	DPP-IV (sol) vs. TyrAP (sol)	0.554	0.026*
	pGluAP (sol) vs. AlaAP (sol)	0.639	0.10*
	pGluAP (sol) vs. ArgAP (sol)	0.986	$1.04*10^{-8}$ ***
	pGluAP (sol) vs. AspAP (sol)	0.986	$1.46*10^{-11}$ ***
	pGluAP (sol) vs. GluAP (sol)	0.937	$2.05*10^{-5}$ ***
	TyrAP (sol) vs. CysAP (sol)	0.585	0.17*
	TyrAP (sol) vs. GluAP (sol)	0.918	$6.65*10^{-5}$ ***
	DPP-IV (mb) vs. AlaAP (mb)	0.549	0.028*
	DPP-IV (mb) vs. CysAP (mb)	0.503	0.047*
	DPP-IV (mb) vs. GluAP (mb)	0.613	0.012*
	pGluAP (mb) vs. AlaAP (mb)	0.813	$1.28*10^{-4}$ ***
	pGluAP (mb) vs. AspAP (mb)	0.991	$1.68*10^{-13}$ ***
	pGluAP (mb) vs. CysAP (mb)	0.975	$1.40*10^{-10}$ ***
	pGluAP (mb) vs. GluAP (mb)	0.919	$4.90*10^{-7}$ ***
Pituitary	DPP-IV (sol) vs. AlaAP (sol)	0.848	$3.30*10^{-5}$ ***
	DPP-IV (sol) vs. AspAP (sol)	0.849	$9.59*10^{-4}$ ***
	DPP-IV (sol) vs. CysAP (sol)	0.733	$1.23*10^{-3}$ **
	DPP-IV (sol) vs. GluAP (sol)	0.700	$2.54*10^{-3}$ **
	DPP-IV (sol) vs. pGluAP (sol)	0.803	$1.83*10^{-4}$ ***
	DPP-IV (sol) vs. TyrAP (sol)	0.574	0.020*
	pGluAP (sol) vs. AlaAP (sol)	0.526	0.036*
	pGluAP (sol) vs. ArgAP (sol)	0.583	0.018*
	pGluAP (sol) vs. AspAP (sol)	0.907	$1.15*10^{-4}$ ***
	pGluAP (sol) vs. GluAP (sol)	0.881	$6.46*10^{-6}$ ***
	TyrAP (sol) vs. AlaAP (sol)	0.637	0.008*
	TyrAP (sol) vs. CysAP (sol)	0.554	0.026*
	DPP-IV (mb) vs. AlaAP (mb)	0.648	$6.63*10^{-3}$ **
	DPP-IV (mb) vs. AspAP (mb)	0.678	$3.93*10^{-3}$ **
	DPP-IV (mb) vs. CysAP (mb)	0.778	$3.91*10^{-4}$ ***
	DPP-IV (mb) vs. pGluAP (mb)	0.800	$1.99*10^{-4}$ ***
	DPP-IV (mb) vs. TyrAP (mb)	0.678	$3.89*10^{-3}$ **
	pGluAP (mb) vs. AlaAP (mb)	0.658	$5.64*10^{-3}$ **
	pGluAP (mb) vs. AspAP (mb)	0.732	$1.26*10^{-3}$ **
	pGluAP (mb) vs. CysAP (mb)	0.986	$2.66*10^{-12}$ ***
	pGluAP (mb) vs. TyrAP (mb)	0.791	$2.64*10^{-4}$ ***
	TyrAP (mb) vs. AlaAP (mb)	0.925	$2.87*10^{-7}$ ***
	TyrAP (mb) vs. ArgAP (mb)	0.674	$4.18*10^{-3}$ **
	TyrAP (mb) vs. AspAP (mb)	0.796	$2.28*10^{-4}$ ***
	TyrAP (mb) vs. CysAP (mb)	0.864	$1.63*10^{-5}$ ***
	TyrAP (mb) vs. GluAP (mb)	0.654	$5.98*10^{-3}$ **
Adrenal	DPP-IV (sol) vs. AlaAP (sol)	0.742	$9.97*10^{-4}$ ***
	DPP-IV (sol) vs. ArgAP (sol)	0.818	$1.07*10^{-4}$ ***

DPP-IV (sol) vs. AspAP (sol)	0.793	$4.17*10^{-4}$ ***
DPP-IV (sol) vs. CysAP (sol)	0.834	$5.94*10^{-4}$ ***
DPP-IV (sol) vs. GluAP (sol)	0.865	$1.53*10^{-5}$ ***
DPP-IV (sol) vs. pGluAP (sol)	0.750	$8.21*10^{-4}$ ***
DPP-IV (sol) vs. TyrAP (sol)	0.710	$2.05*10^{-3}$ **
pGluAP (sol) vs. AlaAP (sol)	0.745	$9.248.21*10^{-4}$ ***
pGluAP (sol) vs. ArgAP (sol)	0.937	$8.96*10^{-8}$ ***
pGluAP (sol) vs. AspAP (sol)	0.953	$3.91*10^{-8}$ ***
pGluAP (sol) vs. CysAP (sol)	0.861	$1.94*10^{-5}$ ***
pGluAP (sol) vs. GluAP (sol)	0.926	$2.65*10^{-7}$ ***
TyrAP (sol) vs. AlaAP (sol)	0.571	0.021*
TyrAP (sol) vs. CysAP (sol)	0.587	0.017*
DPP-IV (mb) vs. AlaAP (mb)	0.840	$4.62*10^{-5}$ ***
DPP-IV (mb) vs. ArgAP (mb)	0.865	$1.53*10^{-5}$ ***
DPP-IV (mb) vs. AspAP (mb)	0.938	$8.26*10^{-8}$ ***
DPP-IV (mb) vs. CysAP (mb)	0.933	$1.35*10^{-7}$ ***
DPP-IV (mb) vs. GluAP (mb)	0.736	$1.14*10^{-3}$ **
DPP-IV (mb) vs. pGluAP (mb)	0.921	$1.11*10^{-6}$ ***
DPP-IV (mb) vs. TyrAP (mb)	0.909	$1.06*10^{-6}$ ***
pGluAP (mb) vs. AlaAP (mb)	0.821	$1.73*10^{-4}$ ***
pGluAP (mb) vs. ArgAP (mb)	0.906	$3.26*10^{-6}$ ***
pGluAP (mb) vs. AspAP (mb)	0.987	$1.28*10^{-11}$ ***
pGluAP (mb) vs. CysAP (mb)	0.971	$1.74*10^{-9}$ ***
pGluAP (mb) vs. GluAP (mb)	0.756	$1.12*10^{-3}$ **
pGluAP (mb) vs. TyrAP (mb)	0.909	$2.79*10^{-6}$ ***
TyrAP (mb) vs. AlaAP (mb)	0.944	$3.77*10^{-8}$ ***
TyrAP (mb) vs. ArgAP (mb)	0.851	$2.92*10^{-5}$ ***
TyrAP (mb) vs. AspAP (mb)	0.924	$3.09*10^{-7}$ ***
TyrAP (mb) vs. CysAP (mb)	0.949	$2.08*10^{-8}$ ***
TyrAP (mb) vs. GluAP (mb)	0.873	$1.01*10^{-5}$ ***

Note: Intratissues Correlation between angiotensinase and stress-regulatory activities that form the hypothalamic-pituitary-adrenal axis. AlaAP: alanyl-aminopeptidase; ArgAP: arginyl-aminopeptidase; AspAP: aspartyl-aminopeptidase; CysAP: cystinyl-aminopeptidase; DPP-IV: dipeptidyl-petidase IV; GluAP: glutamyl aminopeptidase; pGluAP: pyroglutamyl-aminoptidase; TyrAP: tyrosyl-aminopeptidase. Asterisks represent p-value * <0.05, ** <0.01, *** <0.001.

Supplementary Table S6. Significative correlations between central biomarker proline-iminopeptidase (PIP) and regulatory aminopeptidases of the renin-angiotensin system, energy homeostasis of stress and behavior in pituitary and hypothalamus.

Pituitary PIP (fraction) vs.	Aminopeptidase activity (fraction)	Relevance position	Correlation coefficient (R)	P-value
PIP (sol) vs.	DPP-IV (sol)	1	0.863	$1.70*10^{-5}$ ***
	AlaAP (sol)	2	0.861	$1.84*10^{-5}$ ***
	pGluAP (sol)	3	0.771	$4.72*10^{-4}$ ***
	AspAP (sol)	4	0.751	$7.71*10^{-3}$ **
	GluAP (sol)	5	0.647	$6.76*10^{-3}$ **
	TyrAP (sol)	6	0.621	0.010**
	CysAP (sol)	7	0.594	0.015*
PIP (mb) vs.	CysAP (mb)	1	0.987	$1.76*10^{-12}$ ***
	pGluAP (mb)	2	0.984	$6.41*10^{-12}$ ***
	TyrAP (mb)	3	0.835	$5.71*10^{-4}$ ***
	AspAP (mb)	4	0.796	$2.23*10^{-4}$ ***
	DPP-IV (mb)	5	0.752	$7.88*10^{-4}$ ***
	AlaAP (mb)	6	0.710	$2.04*10^{-3}$ **
**Hypothalamus PIP (fraction) vs.**	**Aminopeptidase activity (fraction)**	**Relevance position**	**Correlation coefficient (R)**	**P-value**
PIP (sol) vs.	AlaAP (sol)	1	0.900	$1.99*10^{-6}$ ***
	DPP-IV (sol)	2	0.766	$5.43*10^{-4}$ ***
	AspAP (sol)	3	0.693	$2.93*10^{-3}$ **
	ArgAP (sol)	4	0.690	$3.12*10^{-3}$ **
	pGluAP (sol)	5	0.683	$4.98*10^{-3}$ **
	GluAP (sol)	6	0.660	0.027*
	CysAP (sol)	7	0.651	$6.29*10^{-3}$ **
	TyrAP (sol)	8	0610	0.012*
PIP (mb) vs.	CysAP (mb)	1	0.927	$2.49*10^{-7}$ ***
	AspAP (mb)	2	0.923	$3.46*10^{-7}$ ***
	pGluAP (mb)	3	0.898	$2.41*10^{-6}$ ***
	GluAP (mb)	4	0.880	$7.10*10^{-6}$ ***
	AlaAP (mb)	5	0.642	$7.34*10^{-3}$ **

Note: Correlation of the functional marker of central Proline-iminopeptidase (PIP) activity with angiontensinase and stress regulators activities in soluble pituitary and hypothalamic fractions.
